# Dr. Ayre’s Treatment of Cholera

**Published:** 1854-07

**Authors:** 


### RECORD OF MEDICAL SCIENCE.

*Dr. Ayre's Treatment of Cholera.*

I shall here briefly repeat, for the sake of obviating all misconception on the subject, the leading particulars of what is denominated my plan of treatment, and which consists in the stage of collapse of giving one or two grains of calomel every five or ten minutes, with one or two drops of laudanum with the first few doses of the drug, and in perseveringly continuing the same dose at the same intervals of time, until the symptoms of collapse become materially subdued. This plan I have uninterruptedly pursued from the first to the last patient I ever attended in the disease, amounting to a very large number; and my reason for giving the small dose of the calomel was, that large ones were rejected from the stomach, and I repeated it frequently because it was small, and that thus the action of the medicine on the stomach might be constantly kept up in a disease whose duration is to be counted by minutes. I gave the minute dose of the opiate to enable the stomach to retain the calomel, and prevent its too early descent into the bowels, and not as a sedative. I abstained from the use of all auxiliary treatment in my early cases, that I might not compromise the conclusion at which I desired to arrive—as to the remedial power of calomel; and I have uniformly avoided them since, because I found that calomel in doses small and frequently repeated was the alone remedy. I have never given stimulants in any form, because I found them not to be necessary, and believed they would prove pernicious when, from the long duration of the collapse, and the delay in commencing the treatment, consecutive fever might be feared; and lastly, I fixed no limit to the quantity of calomel which I gave than that which the duration of the collapse prescribed, having become early assured that pending its continuance no absorption of the calomel into the system takes place, and that whilst it is so given, no salivation or other inconvenience is induced by it, and that no extremity to which a patient may be reduced can justify our withholding or abandoning the use of it.—*Letter from Dr. Ayre, of Hull, to the President and Fellows of the Royal College of Physicians. (London Lancet.)*

